# Challenges and opportunities in NMIBC management across Latin America: insights from healthcare providers and a patient advocacy group

**DOI:** 10.3332/ecancer.2024.1711

**Published:** 2024-06-07

**Authors:** Mark McCully, Julia Lipkis, Aryel Heller, Adrian Huñis

**Affiliations:** 1 PharmaValue Partners, 603 Mattison Avenue, Suite 319, Asbury Park, NJ 07712, USA; 2 A. Hunis & Associates, Oncology Consultants, Hollywood, FL 33019, USA

**Keywords:** NMIBC, patient advocacy, BCG, clinical trials, Latin America, urology, public health, Brazil, Colombia, Mexico, Argentina

## Abstract

Non-muscle invasive bladder cancer (NMIBC) is characterised by high rates of recurrence and progression, requiring substantial healthcare resources. In Latin America, the incidence of NMIBC is set to increase due to an aging population and lifestyle changes. To better understand the current challenges for NMIBC treaters and patients, a mixed-methods approach was leveraged combining secondary research with qualitative interviews from healthcare providers in Brazil, Colombia, Mexico and Argentina. Our analysis found that significant challenges persist across the region, particularly due to *Bacillus* Calmette-Guérin shortages, inconsistent adherence to clinical guidelines and significant socioeconomic disparities for patients accessing healthcare services. Addressing these challenges requires improved patient advocacy, strategic use of clinical trials and better resource distribution to enhance NMIBC management across Latin America.

## Background

Non-muscle invasive bladder cancer (NMIBC) is a treatable yet challenging form of bladder cancer, known for its high rates of recurrence and progression which require continuous monitoring and significant healthcare resources [1–4]. It has been reported that bladder cancer costs were associated with the highest per-patient cost of all cancers from diagnosis to death [1]. In the United States, a recent report by Williams et al [5], estimated that in 2020 treatment costs for bladder cancer approached nearly 6 billion USD, with median costs escalating from 29,459 USD in the first year to 55,267 USD in the second year, and reaching 117,361 USD by the fifth year following the start of Bacillus Calmette-Guérin (BCG) induction.

Latin America’s diverse healthcare systems pose significant and substantial challenges to effectively treat and manage NMIBC [6–17]. Moreover, the incidence of bladder cancer in the region is expected to increase over the coming years due to an aging population and changes in lifestyle [1].

The challenges of managing NMIBC in Latin America are multifaceted. One of the most pressing issues is the worldwide shortage of BCG, the primary treatment for high-risk NMIBC cases. Throughout Latin America, BCG is generally in low supply, which leads to its use being restricted to high-risk patients [18–20]. While chemotherapy can be used in the absence of intravesical BCG, its availability varies across countries. This shortage is compounded by varying standards of care, healthcare infrastructure constraints and significant disparities in access to healthcare services across urban and rural areas.

Additionally, lifestyle factors significantly influence the prevalence and management of NMIBC [21]. Smoking is closely linked with bladder cancer, and the prevalence of smoking in the region varies significantly by country, affecting disease incidence rates and complicating public health efforts to reduce cancer risks [1, 2]. Dietary factors also play a role, with changing diets potentially leading to higher rates of diseases previously less common in the region [2].

Finally, the quality of urology training programs in Latin America varies significantly, with a third of urology residents not receiving objective accreditation tests upon completion of their programs, and many lacking exposure to advanced surgical techniques and procedures [22]. Based on all these disparities, we selected four Latin American countries to explore the specific challenges and opportunities facing NMIBC patients and healthcare providers.

## Methods

Utilising a mixed-methods approach, this paper integrates comprehensive secondary research with primary qualitative data gathered from interviews across four Latin American countries: Brazil, Colombia, Mexico and Argentina (Table 1). A review of existing literature and data was first performed to contextualise the current landscape of NMIBC care, as well as to inform primary interview questions and support subsequent findings. Primary interviews were conducted with healthcare providers experienced in the diagnosis and treatment of NMIBC patients, as well as with a representative from a major patient advocacy group who highlighted major challenges and barriers from a patient perspective navigating NMIBC diagnosis and care in Latin America. Each interview aimed to capture detailed insights into the current practices, challenges and perceptions regarding NMIBC management.

## Results

### Brazil

#### Standard of care

Despite a relatively well-structured healthcare system, adherence to NMIBC guidelines is somewhat inconsistent, primarily due to BCG supply issues [15]. Brazilian urologists typically follow the European Association of Urology (EAU) guidelines, but full adherence may vary, especially when treating patients with recurrent NMIBC. According to secondary sources, only about two-thirds of NMIBC treaters adhere to these guidelines post-BCG failure, due to persistent shortages [23]. Other challenges include limited access to chemotherapy agents as well as high costs, which can lead to deviations from recommended treatments [16, 21].

#### BCG use

Brazil faces significant BCG shortages, largely due to the recent closure of domestic manufacturing facilities over regulatory issues. As a result, hospitals rely on imported BCG, which is often in short supply [24].

#### Patient access

The patient journey from symptom onset to diagnosis is often a lengthy process, particularly in the public sector, where delays can compromise treatment outcomes [16]. Access to diagnostic and treatment facilities varies between 6 months and 2 years, with private patients generally receiving faster and more comprehensive care. Healthcare providers express frustration with cultural and systemic barriers, such as hesitancy to seek initial care and long waiting lists, that lead to delayed diagnosis.

#### Healthcare provider education

Brazilian urologists typically keep themselves updated on advances in the field through international guidelines, participation in congresses and ongoing professional education, indicating general engagement with both current practices and emerging research in their field. NMIBC treaters express interest in novel immunotherapies; however, high costs and limited clinical trials within the country slow the widespread adoption of newer therapies.

### Colombia

#### Standard of care

Colombia exhibits significant variability in the management of NMIBC, largely influenced by geographic disparities in healthcare access. Adherence to international guidelines is inconsistent, especially in rural areas where healthcare resources are sparse. Urban centers like Bogotá have better infrastructure and are more likely to follow these guidelines closely [25]. Challenges include a severe shortage of BCG and a lack of trained urologists, which can delay or alter the standard treatment protocol [26, 27].

#### BCG use

Colombia faces acute shortages of BCG, more severe than in many other countries, affecting the standard care for NMIBC patients. The only BCG product with an approved health registration, ‘SII Onco BCG,’ is imported in limited quantities, and the supply does not meet the patient demand, leading to significant treatment delays and the use of alternative therapies where feasible [28].

#### Patient access

Patients in Colombia often experience lengthy delays from symptom onset to diagnosis and treatment, especially within the public health system. These delays are exacerbated by insufficient healthcare infrastructure and a centralised healthcare system that does not adequately serve rural and remote areas. Patient navigation is hindered by complex referral systems and limited access to specialised care.

#### Healthcare provider education

Healthcare professionals in Colombia face challenges in accessing the latest training and resources, particularly outside major urban centers. While there is interest in advancing NMIBC treatment through novel therapies and techniques, actual implementation is slow due to these educational gaps. Urologists and oncologists strive to stay informed through limited participation in international conferences and digital learning platforms.

### Mexico

#### Standard of care

In Mexico, the approach to NMIBC care is relatively comprehensive in urban areas but is highly inconsistent across the country due to the uneven distribution of healthcare resources. Most major cities have adequate facilities to follow EAU guidelines, but rural areas often have insufficient access to specialised care, leading to significant deviations from these guidelines [17, 29].

#### BCG use

Mexico does not possess any local production centers for BCG and therefore depends upon imported strains of the virus. Shortages often occur which lead to distribution issues, forcing healthcare providers to ration BCG use or switch to alternative treatments.

#### Patient access

The disparity in healthcare access between urban and rural areas significantly affects the NMIBC treatment pathway in Mexico. Urban centers provide relatively quick access to diagnosis and treatment, while rural patients may face long travel times and delays in receiving care, impacting treatment outcomes.

**Healthcare provider education**

Mexican healthcare providers are generally well-engaged with the latest developments in NMIBC care, participating in national and international medical conferences and professional societies. However, the reach of this education may vary, with practitioners in remote areas having less access to ongoing education and resources [30].

### Argentina

#### Standard of care

Argentina’s healthcare system provides relatively uniform access to NMIBC care, and most treaters are well-versed in international guidelines [11, 31, 32].

#### BCG use

The national INPB Mycobacteria Derivatives Service produces BCG domestically, which helps ensure better availability than in many surrounding countries. Despite this local production, shortages and distribution challenges still sometimes occur, leading to the rationing of BCG, especially during maintenance therapy [6, 33].

#### Patient access

While Argentina boasts a robust healthcare infrastructure, some disparities exist between urban and rural areas in terms of speed and quality of NMIBC care. Urban patients typically experience faster diagnosis and treatment initiation compared to those in rural areas, where healthcare resources are more limited.

#### Healthcare provider education

Argentinian healthcare professionals are active in both national and international urological communities, which contributes to a high level of knowledge and competency in NMIBC care. There is significant interest in integrating novel therapies into practice, although practical implementation can be hindered by regulatory and cost-related challenges.

#### Cross-country comparison and discussion

Table 2 provides a comparative analysis of the incidence, resources and healthcare practices related to NMIBC management in Argentina, Brazil, Colombia and Mexico.

## Discussion

The management of NMIBC in Latin America is fraught with challenges, including widespread BCG shortages, inconsistent adherence to clinical guidelines and significant geographic and socioeconomic disparities. These issues, while prevalent across Argentina, Brazil, Colombia and Mexico, impact each country differently, influencing both patient outcomes and the overall effectiveness of NMIBC management.

A primary concern in the region is the scarcity of intravesical BCG, the fundamental treatment for high-risk NMIBC [18, 19, 21]. Brazil, once a producer of the Moreau BCG strain, has encountered severe supply disruptions. Production ceased due to compliance issues with good manufacturing practices (GMPs) and delays in establishing a new facility. Consequently, Brazil has had to import BCG at significantly higher costs, forcing hospitals to independently manage their BCG supplies. This has led to inconsistent treatment availability and compromised patient care [24].

In Colombia, the availability of BCG is critically low, with only one approved product [28]. High import costs and logistical challenges compound this issue, severely limiting access to essential treatments and necessitating that healthcare providers prioritise BCG for the most at-risk patients, often to the detriment of comprehensive care.

Argentina and Mexico also face significant BCG supply issues, though their challenges differ. Argentina struggles with production inconsistencies despite having manufacturing facilities, resulting in only moderate availability. In Mexico, vast geographic and economic inequalities exacerbate the shortage, particularly affecting rural areas where adherence to treatment guidelines is low, and patient outcomes vary significantly [29].

Healthcare disparities, particularly in rural and remote areas, limit access to well-trained medical personnel and infrastructure, thereby impacting NMIBC care. Investment in healthcare infrastructure, training programs and targeted initiatives can improve care in underserved areas.

These disparities in guideline adherence reflect broader issues of resource availability and the need to resort to alternative treatments, which are often less effective [21]. The role of patient advocacy is emerging but remains limited across the region. Advocacy groups, often focused on more common cancers, may overlook NMIBC given its lower incidence in the region. However, these groups could potentially help NMIBC survivors to establish their own NMIBC-specific advocacy initiatives, leveraging their unique experiences for support, public engagement and policy influence.

By addressing the unique challenges of each country with targeted policies and leveraging regional strengths, Latin America can improve NMIBC care outcomes and reduce the disease’s burden. Enhanced awareness, improved resource distribution and strategic use of clinical trials could become crucial for advancing NMIBC management in this diverse and dynamic region.

Based on our secondary and primary research we propose the following regional strategies to foster better NMIBC care:

1. **Enhanced patient advocacy:** Throughout the LATAM region, patient advocacy groups tend to be pan-tumor with more frequently occurring cancers prioritised. The establishment of NMIBC-specific advocacy groups across Latin America could lead to greater awareness of this increasingly prevalent cancer, resulting in the development of resources to help patients navigate a complex treatment journey, while also influencing country-specific health policies. As an example, patient advocacy groups might be able to lobby governments to subsidise BCG costs and expedite new treatment approvals.
2. **Harmonised clinical trial regulations:** As mentioned previously, the high level of regulatory diversity in the LATAM region impacts the running of clinical trials in the region as well as the approval of new medicines. More standardised clinical trial regulations could facilitate multi-country studies, speed up the approval process for new treatments and address BCG shortages. Using Colombia as an example, the government facilitated highly favorable clinical trials and R&D tax incentives to bring Colombia’s clinical research environment up to international standards and enhance its relative attractiveness. As reported, experts agree that Colombia could see over 100 new clinical trials every year and close to $500 million in economic gains per year [34]. By creating a harmonised regulatory environment for clinical trials, patients within these regions could get access to cutting-edge treatments years earlier.
3. **Incentives for trial sponsor:** Leveraging incentives such as tax breaks and fast-track approval processes for pharmaceutical companies conducting trials or investing in research within the region could accelerate access to novel NMIBC drugs and improve patient outcomes through increased resources and clinical education within the region [34].
4. **Education and training programs:** Initiate comprehensive education campaigns targeted at both healthcare providers and patients to enhance understanding of NMIBC, improve treatment compliance and promote guideline adherence. Enhance patient awareness programs to improve understanding of NMIBC treatment pathways and the importance of guideline adherence.
5. **Infrastructure investment and localised patient recruitment:** Promote clinical trials in rural and underserved regions to increase access to innovative treatments and enhance patient compliance by providing care closer to home. Develop mobile health clinics and telehealth services to extend reach in underserved areas [13].

## Conclusion

Latin America faces significant challenges managing NMIBC due to complex and heterogeneous healthcare provisions. These challenges are further exacerbated by routine BCG supply shortages, disparities in healthcare access, lack of patient awareness about NMIBC and variability in guideline compliance. Addressing these issues requires a multifaceted approach that engages healthcare providers, governments, pharmaceutical companies and patient advocacy groups.

## Key Challenges to Address

1. **Healthcare disparities and resource distribution:** Tackling disparities in healthcare access, especially in rural and remote areas, necessitates investments in infrastructure, professional training programs and initiatives that improve care delivery in underserved regions. Efficient distribution of resources – such as healthcare facilities, medical supplies and skilled personnel – is crucial to ensure equitable NMIBC care. Strategic planning and stakeholder collaboration will optimise resource allocation and help bridge the gap in healthcare access across different regions.
2. **BCG supply shortages:** BCG therapy, the standard treatment for NMIBC, is often in short supply globally, but shortages are uniquely pronounced in Latin America. This issue can be mitigated through diversification of suppliers, strategic stockpiling and international cooperation to ensure a consistent BCG supply.
3. **Lack of patient awareness:** Many patients in Latin America remain unaware of NMIBC symptoms, risk factors and treatment options. Public awareness campaigns, educational programs in healthcare facilities and partnerships with patient advocacy groups can promote early detection and timely treatment.
4. **Variability in guideline compliance:** Adherence to NMIBC diagnosis and treatment guidelines varies across healthcare providers and regions. Standardising protocols, additional training for healthcare professionals and implementing quality assurance measures can improve compliance and ensure high-quality care.
5. **Tailored policies and strategies:** Each country in Latin America has unique healthcare challenges and resources. Policies and strategies should be tailored to address specific needs while leveraging regional strengths, such as established research institutions and healthcare networks, to optimise NMIBC management.
6. **Strategic use of clinical trials:** Clinical research and trial participation can advance NMIBC management by evaluating new treatment methods, refining existing therapies and generating guidelines tailored to regional needs. Collaboration with academic institutions, pharmaceutical companies and regulatory agencies can facilitate trial conduct.

By implementing targeted policies and strategies tailored to each country’s unique challenges while harnessing regional strengths, Latin America can significantly enhance NMIBC care outcomes and reduce the disease’s impact across the region. Promoting greater awareness, optimising resource distribution and strategically utilising clinical trials will be vital to advancing NMIBC treatment throughout this diverse and dynamic area.

## Conflicts of interest

The authors report no conflicts of interest.

## Funding

Support for the research was provided by a Pfizer Global Medical Grant (GMG).

## Figures and Tables

**Table 1.** Respondent information.

Country	Specialty	Sub-specialty	Years in practice
Argentina	Urology	Oncology	15
Brazil	Urology	Oncology	9
	Urology	Robotic surgery	6
Colombia	Urology	Oncology	5
	Director patient advocacy foundation	-	-
Mexico	Urology	Laparoscopic surgery	12

**Table 2.** NMIBC SOC cross-country comparison [3].

	Argentina	Brazil	Colombia	Mexico
Bladder cancer incidence (ASR)	5.2	5.4	2.8	2.5
Adherence to NMIBC guidelines	High	Moderate	Low	Low
BCG availability	Moderate	Low	Very Low	Low
BCG local production	1 facility	Paused due to GMP issues	N/A, imported	N/A, imported
BCG usage strategy	1 year of maintenance for high-risk patients	Rationed to high-risk patients, alternatives used	High-risk patients prioritised when possible	Full-strength for high-risk patients, alternatives for others
